# Justice, Disability, and Law: A Neurological and Ethical Assessment of Multiple Sclerosis in Turkey

**DOI:** 10.1186/s12910-026-01434-2

**Published:** 2026-03-17

**Authors:** Furkan Yılmaz, Furkan İncebacak, Hakan Acar, Abdullah Güzel

**Affiliations:** 1 Department of Neurology, Afyonkarahisar State Hospital, Afyonkarahisar, Turkey; 2 Department of Neurology, Faculty of Medicine, Afyonkarahisar Health Sciences University, Afyonkarahisar, Turkey; 3 Department of Neurology, Bursa Çekirge State Hospital, Bursa, Turkey

**Keywords:** Empirical bioethics, Disability justice, Multiple sclerosis, Judicial decision-making, CRPD, Neurocognitive assessment

## Abstract

**Background:**

This study analyzes Supreme Court decisions involving parties diagnosed with Multiple Sclerosis (MS) between 2008 and 2024, focusing on disability justice and the normative evaluation of judicial practice. MS is a chronic neurological disease with potential physical and neurocognitive consequences that may affect legal capacity, employment, and social participation. The study is grounded in the United Nations Convention on the Rights of Persons with Disabilities (CRPD), Daniels’ principle of Fair Equality of Opportunity, and Fricker’s concept of epistemic injustice.

**Methods:**

A qualitative document analysis was conducted on 85 Supreme Court decisions in which at least one party had an MS diagnosis. Decisions were classified according to civil or criminal chambers and type of legal dispute. The analysis examined reliance on institutional medical reports, requests for individualized neurological or neurocognitive assessments, and the compatibility of judicial reasoning with normative disability justice standards.

**Results:**

Of the 85 decisions, 63 belonged to civil chambers and 18 to criminal chambers (HGK: 3, CGK: 1). In civil law, divorce cases were most frequent (24/63; 38.1%), followed by disability or social security disputes (10/63), labor law cases (7/63; 11.1%), and work accident or workforce loss cases (6/63). In disability or workforce loss cases (*n* = 16), Social Security Institution reports were considered decisive in 14 cases (87.5%), while independent neurological evaluations were requested in only three cases. In criminal cases (*n* = 19), individuals with MS were victims in 12 cases and defendants in 7; forensic medical reports were obtained in 5 of the defendant cases, with partial criminal capacity reduction applied in only one case. No explicit reference to standardized neurocognitive testing (e.g., MoCA, MMSE, ACE-R) was identified in the publicly accessible decision texts.

**Conclusions:**

The frequent prioritization of institutional reports over individualized neurological assessments, together with the limited consideration of neurocognitive vulnerability, may be in tension with Articles 12 and 27 of the CRPD and may contribute to structural inequality. To promote disability justice, we propose adopting personalized neurological assessment as a procedural minimum standard, ensuring independent expertise in administrative–clinical report conflicts, clarifying reasonable accommodation obligations in labor law, introducing aggravating circumstances in crimes against disabled victims, and incorporating standardized neurocognitive tests into criminal capacity evaluations.

## Introduction

The global number of Multiple Sclerosis (MS) patients has approached 2.8 million, showing an upward trend in all World Health Organization regions [1]. Physically, approximately 80% of cases develop limitation in gait and mobility within 10–15 years of diagnosis; some of them require mobility aids [2, 3]. Approximately 41% of individuals with MS have cognitive impairment; it is around 32.5% (95% CI: 29.3–36.0) in the relapsing–remitting form and about 50–70% in progressive types [4, 5].

MS is a disease with not only medical but also social and legal dimensions due to its chronic course and neurocognitive effects. Disability has been redefined as a matter of rights with the United Nations Convention on the Rights of Persons with Disabilities (CRPD) dated 2006; positive obligations have been imposed on states in the fields of equality in working life, access to justice and social security [6–9]. The European Court of Human Rights has also recognized equal access to health and employment for persons with disabilities as a protected right and has imposed responsibility on states in violations [10].

For individuals with MS, this normative framework has significant implications for both employment and social security processes [8]. Various studies show that individuals with MS may be discriminated against in the world of work, their demands for “reasonable accommodation” are met in a limited way, and economic losses are widespread [2, 11–13]. The European Court of Human Rights (ECtHR) has also recognized equal access to health as a protected right for persons with disabilities and has imposed responsibility on states for violations [10].

In Turkey, studies on judicial decisions on disability are limited. Analysis of previous Supreme Court (Court of Cassation) decisions on injection-induced sciatic nerve injuries has shown that court records can be a valuable data source for public health and patient safety [14]. Similarly, this study aims to evaluate the practices at the intersection of disability, health, and law by systematically examining the decisions of the Court of Cassation involving individuals diagnosed with MS.

This study analyzes 85 decisions involving parties diagnosed with MS in the Court of Cassation between 2008 and 2024; It aims to classify structural disadvantage patterns that arise in the fields of social security, labor, family, and criminal law. The findings will be compared with international normative frameworks such as ADA, CRPD and ECtHR case-law to assess the gaps between practice in Turkey and global standards of disability rights. In line with the results obtained, it is aimed to develop evidence-based policy recommendations to strengthen justice and health equity for individuals with MS.

This manuscript adopts an explicit descriptive-normative structure commonly used in empirical bioethics. The descriptive component maps how multiple sclerosis (MS) functioned procedurally across Supreme Court case types (e.g., disability determination, employment disputes, family law, and criminal proceedings), with particular attention to expert evidence practices and the visibility of neurocognitive vulnerability in judicial reasoning. The normative component then evaluates these descriptive patterns against disability justice standards, primarily the CRPD, Daniels’ Fair Equality of Opportunity, and Fricker’s epistemic injustice, aiming to identify feasible procedural minimum standards and policy options. Importantly, our normative analysis is framed as an assessment of alignment and risk rather than a determination of illegality in individual cases.

## Materials and methods

In this retrospective document analysis, the data were obtained from anonymized decision texts published publicly in the Decision Search System of the Presidency of the Court of Cassation. The research was conducted to cover the decisions between January 1, 2008 and December 31, 2024; “multiple sclerosis”, “MS” and spelling variations were used in searches. In the published decision texts, the files in which the diagnosis of Multiple Sclerosis (MS) had an impact on the course of the dispute were included in the analysis; only decisions in which the MS expression is referred to or exemplary or whose access is restricted in the system are excluded. Descriptive information regarding names, institution titles and file numbers in the decision texts have been anonymized in accordance with the Personal Data Protection Law of Türkiye (PDPL).

### Data extraction and coding

We developed a structured extraction form and codebook through (i) an a priori framework informed by disability justice concepts (CRPD; Fair Equality of Opportunity; epistemic injustice) and (ii) iterative refinement after pilot coding. Core coding categories captured: (1) chamber/assembly and year; (2) case type (civil/criminal and sub-type); (3) party role of the person with MS (e.g., plaintiff, defendant, victim); (4) appellate outcome (affirmance, reversal, partial reversal) and reasoning pattern; (5) procedural role of MS in the dispute (e.g., disability/work capacity determination, accommodation/dismissal, economic vulnerability in family law, causation/damages, victim vulnerability, criminal responsibility/competence); (6) type of medical/expert evidence referenced (e.g., SSI/SGK, Forensic Medicine Institute/ATK, hospital board, independent neurologic or neuropsychological assessment); and (7) whether individualized neurologic or neurocognitive assessment was explicitly requested or discussed. Two researchers coded all texts independently; disagreements were resolved by discussion and adjudication with a third researcher. Coding and descriptive tabulation were performed in Microsoft Excel. This study is descriptive; advanced statistical tests were not applied. Ethics committee approval was not required because publicly available judicial records were used and personal data were anonymized.

## Results

A total of 85 (*n* = 85) decisions were examined. Most of the cases are in civil chambers: 63 (74.1%) are civil chambers; 18 (21.2%) criminal chambers; 3 (3.5%) General Assembly of Civil Chambers; 1 (1.2%) Criminal General Assembly. The total number of files in the penal area is 19 (Criminal Chambers 18 + Criminal General Assembly 1) (Table 1).

**Table 1:** Distribution of Supreme Court Chambers / General Assemblies (*n* = 85)

Chamber / Assembly	Number of Cases	% (*n* = 85)
Civil Chambers	63	74.1
Criminal Chambers	18	21.2
Civil General Assembly (HGK)	3	3.5
Criminal General Assembly (CGK)	1	1.2

Civil chambers (*n* = 63). Divorce ranks first with 24 decisions (38.1%). A total of 16 decisions (25.4%), including 10 disability-social security and 6 occupational accidents/loss of workforce, constitute the second largest cluster. 7 decisions in labor law (11.1%); the remaining 16 decisions (25.4%) are in areas such as inheritance, trade, compensation and enforcement (Table 2).

**Table 2:** Distribution of Civil Cases by Type (*n* = 63)

Type of Civil Case	Number of Cases	% (*n* = 63)
Divorce	24	38.1
Disability / Social Security	10	15.9
Occupational Accident / Loss of Work Capacity	6	9.5
Labor Law	7	11.1
Other (inheritance, commercial, compensation, enforcement)	16	25.4

Disability and labour loss files (*n* = 16). In 14 (87.5%) of the files, the courts accepted the SSI report as decisive; Additional independent neurological evaluation was requested in 3 files (in some cases, the two approaches were applied together). The procedures and criteria of disability determination in Turkey are regulated by the relevant regulation [15] (Table 3).

**Table 3:** Disability and Work Capacity Cases (*n* = 16)

Indicator (categories may overlap)	Number of Cases	% (*n* = 16)
Social Security Institution (SSI/SGK) report accepted as decisive	14	87.5
Independent neurological reassessment requested	3	18.8

Labor law (*n* = 7). In five of the seven files (71.4%), the decision was concluded in favor of the employer; claims of dismissal or reasonable accommodations were mostly rejected.

Family law/divorce (*n* = 24). Permanent alimony was ruled in only 6 files (25%); The acceptance rate is significantly lower in cases where men with MS are defendants.

Penalty area (total *n* = 19). In 12 files (63.2%), the person with MS was the victim; the most common crimes were sexual assault and theft/looting. In 7 files (36.8%), the person with MS was the defendant (Table 4). Among these defendant cases, a Forensic Medicine (ATK) report was obtained in five (71.4%), but a partial criminal responsibility reduction was applied in only one case (14.3%) (Table 5). Only one file included a partial driver’s license restriction. Standardized neurocognitive tests were not documented in the publicly accessible decision texts.

**Table 4:** Criminal Cases: Role Distribution (*n* = 19)

Role of the Person with MS	Number of Cases	% (*n* = 19)
Victim	12	63.2
Defendant	7	36.8

**Table 5:** Defendant Subset (*n* = 7)

Criminal Defendant Cases	Number of Cases	% (*n* = 7)
Forensic Medicine (ATK) report obtained	5	71.4
Partial criminal responsibility reduction applied	1	14.3

## Discussion

This study is based on the evaluation of 85 public decisions reviewed by the Supreme Court between 2008 and 2024, in which one of the parties was diagnosed with Multiple Sclerosis (MS). The aim is to examine the practices that have emerged at the intersection of disability, health and law in Turkey in general terms. In practice, disability in legal proceedings is often determined according to institutional reports; in labor disputes, requests for “reasonable accommodation” are addressed only in a limited way; long-term economic disadvantage may appear as a secondary element in divorce cases; and in criminal matters, victim vulnerability and the defendant’s neurocognitive status are infrequently documented with standardized tests. Taken together, these findings raise concerns regarding alignment with the CRPD’s principles of equal recognition before the law (Art. 12) and access to employment (Art. 27), the employment protections under the Americans with Disabilities Act (ADA), and the ECtHR’s disability-related case-law [8, 10, 12].

### Descriptive findings: patterns in evidence and reasoning

Across civil and criminal adjudication, MS-related issues commonly appear as technical questions—such as disability percentage, work capacity, neurocognitive status, or criminal responsibility—that are primarily addressed through expert evidence. In the reviewed case types, MS assumed distinct procedural roles: (i) disability and work-capacity determination in social security and compensation disputes; (ii) accommodation and dismissal arguments in employment litigation; (iii) assessment of economic vulnerability and future care needs in family law disputes; and (iv) consideration of victim vulnerability, competence, and criminal responsibility in criminal proceedings. These differentiated roles shaped both the evidentiary focus and the doctrinal framing of appellate reasoning.

In disability and work-capacity disputes, MS typically forms the central factual premise of the claim, determining eligibility criteria, disability percentage, or workforce loss. Such matters are treated as technical assessments and are therefore largely mediated through medical documentation and expert reports—most frequently institutional Social Security Institution (SSI/SGK) materials, sometimes supplemented by hospital boards or renewed evaluations. In employment litigation, MS is procedurally relevant through dismissal justifications and the feasibility of workplace adjustments. The evidentiary record generally includes medical documentation and job descriptions; however, structured documentation of an interactive accommodation process is rarely visible in appellate reasoning. In family law disputes, MS is not usually the direct object of proof but becomes relevant to earning capacity, economic vulnerability, and anticipated care needs. Here, the evidentiary focus often centers on income documentation and general health status, with limited visibility of individualized neurologic or neurocognitive assessment in the accessible texts. In criminal proceedings, where the person with MS is a victim, the diagnosis may function as a vulnerability factor influencing access-to-justice considerations and sentencing reasoning; where the person with MS is a defendant, the key procedural issue concerns competence and/or criminal responsibility. Although expert evidence—frequently from the Forensic Medicine Institute (ATK)—plays a pivotal role in this context, explicit documentation of standardized neurocognitive testing remains uncommon in publicly available reasoning.

When the international literature on disability and workforce loss is examined, it is concluded that evaluating disability only with medical or administrative rates may under-reflect the real level of functioning and the person’s situation; ICF-compatible functional and cognitive measures are suggested to partially close this gap, (CRPD Articles 12 and 27) [8, 16–18]. In the disability and workforce loss files examined, the courts mostly accepted Social Security Institution (SSI) reports as decisive and, as far as could be understood from the decision texts, independent neurological evaluations were included only in certain cases. In a decision of the 10th Civil Chamber (Case ID 61), the appellate reasoning relied primarily on an institutional SSI disability report, despite the presence of cross-system discrepancies in disability percentage. The publicly accessible reasoning did not document a detailed individualized neurological reassessment beyond reference to the institutional report. This suggests that standardized tools capturing MS-specific domains such as information-processing speed and attention (e.g., Symbol Digit Modalities Test [SDMT], Paced Auditory Serial Addition Test [PASAT]) are not routinely represented in decision processes [19–21]. Reasoned weighing of institutional-report vs. clinical-report contradictions and the routine use of functional tests can provide an ethically defensible basis for evidence-based justification and procedural equity [22].

Empirical studies conducted under the ADA indicate that discrimination claims by employees with MS are relatively frequent and that the duty of “reasonable accommodation” is often interpreted narrowly [11, 12]. Because neurocognitive effects—such as fatigue, slowed processing speed, and fluctuating cognitive performance—are less visible than physical disabilities, accommodation requests for these effects are often inadequately addressed [5, 13]. The fact that five of the seven labor law cases examined in this study concluded in favor of the employer, and that measures such as task redesign, flexible hours, scheduled breaks, or low-stimulus work environments were either not mentioned or were only briefly considered in the reasoning, suggests that the employer’s reasonable accommodation duty may not have been fully operationalized in practice. In a decision of the 22nd Civil Chamber (Case ID 53), the dismissal of an employee following an MS diagnosis was upheld. While medical documentation was acknowledged, the reasoning did not document a structured interactive accommodation process (e.g., reassignment, task modification, or proportionality analysis), nor did it reflect a detailed “undue hardship”-type evaluation. Supporting equality and disability provisions in Turkish legislation with objective criteria akin to the ADA’s “undue hardship” test—and distributing the burden of proof more evenly between the parties—could offer a more inclusive framework for equal access to employment for persons with disabilities [8, 15, 23, 24].

The progressive nature of multiple sclerosis can significantly impact income continuity, sharing of roles within the family, and psychological balance due to the need for care, which can increase over time. It is emphasized in studies that the risk of divorce increases and income decrease due to loss of workforce is more common, especially in male MS patients [2, 25–27]. The fact that alimony was awarded in only six of the 24 divorce cases examined in this study and the low rate of decisions in favor of men with MS as defendants suggests that alimony evaluations may not always be sufficiently sensitive to the effects of the disease over time. Instead of “static” criteria based on fixed income level, the use of more dynamic and disease-sensitive parameters that take into account the risk of loss of function and care and treatment expenses that may develop due to their progressive nature; additionally, incorporating social assistance mechanisms such as care allowances or long-term rehabilitation support into the reasons for decisions can provide a more holistic approach that can support equal opportunities in the long run [17, 18].

From the perspective of criminal cases, the approach of the European Court of Human Rights and Article 16 of the Convention on the Rights of Persons with Disabilities (CRPD) clearly define the positive obligations of the state in protecting persons with disabilities against violence, abuse and exploitation (8, art. 16). However, in many countries, even where the victim’s disability facilitates the commission of the offence, this vulnerability is not consistently taken into account in sentencing [10]. Considering that the victim was diagnosed with MS in 12 of the 19 criminal cases examined in this study and that the most common offences were sexual assault and theft/robbery, disability can create a “facilitating context” for the perpetrator. Yet the fact that victim vulnerability appears only as a limited aggravating factor in the reasons available may be considered limited with respect to both deterrence and an inclusive understanding of justice. Addressing vulnerability more explicitly at sentencing and updating the normative framework where needed can yield closer alignment with CRPD and ECtHR standards (8, arts. 13 & 16). On the other hand, although the effects of neurological disease on decision-making, attention and impulse control are well documented, these domains are often considered only qualitatively in criminal responsibility assessments and standardized testing is used sparingly [28, 29]. In a decision of the 12th Criminal Chamber (Case ID 43), the reasoning referenced clinical considerations related to medication effects in a traffic-related incident involving a defendant diagnosed with MS. However, standardized neurocognitive instruments (e.g., MoCA, MMSE, SDMT, or BICAMS components) were not documented in the publicly accessible decision text, and the assessment relied primarily on general clinical impressions. In our material, despite the Forensic Medicine Institute report being obtained in five of the seven files, only one showed a partial driving-licence restriction; where visible, decisions did not cite structured neuropsychological measures such as SDMT (or BICAMS components). Incorporating validated cognitive tools and providing reasoned interpretations can strengthen evidence-based justification, procedural equality, and the predictability of decisions [19, 20, 22].

When the evaluated areas are taken together, it is seen that courts mostly rely on institutional reports, while personalized medical and neurological evaluations are less covered. This may lead to individual characteristics not being adequately reflected in decision-making processes and the principle of equal opportunity not being fully ensured. The use of independent neurological assessment and standardized tests as the main method in disability files; defining the obligation of “reasonable accommodation” in labor cases with clear and measurable rules; taking into account the sensitivity and need for care related to illness in family law; In criminal law, the explicit inclusion of both the victim’s vulnerability and the defendant’s neurocognitive state in reasoned decisions can contribute to making the judicial process fairer and more sensitive.

### Normative evaluation: CRPD, Daniels, and Fricker

From an ethical perspective, Daniels’ “Fair Equality of Opportunity” approach does not limit equality to everyone being treated the same; emphasizes that creating supportive mechanisms for health-related limitations is also a part of justice (Daniels, [22]). This understanding aims to provide support at a structural level to address the challenges individuals face in real life. The concept of “knowledge-based injustice” put forward by Fricker draws attention to the deficiencies that may be created in terms of justice if the experience and knowledge of persons with disabilities may not always be adequately taken into account in decision-making processes (Fricker, [30]). When these perspectives are considered together with CRPD’s autonomy and dignity-based approach, it is important to ensure that personal medical and neurocognitive information is included in judicial processes in an orderly and transparent manner; In addition, a clear and systematic evaluation of reasonable accommodations and justifications for victim vulnerability can provide a more ethically balanced and human-sensitive approach.

### Recommendations: procedural minimum standards

In line with the results of this study, some improvement suggestions that can be implemented can be summarized as follows:

(A) Harmonizing the Social Security Institution (SSI) legislation with the CRPD and ICF principles in disability assessments; creating standard assessment forms in which cognitive tests and functional criteria are clearly defined; and when there is a difference between institutional reports and clinical opinions, it may be useful to explain the reasons for this in a reasoned manner.
(B) Keeping a written record of the “interaction process” in which the needs of the employee, the requirements of the job and the employer’s possibilities are evaluated together in terms of protective arrangements in the world of work; discussing the arrangements that can be adapted (e.g. scheduled breaks, change of duties, quiet working area, shift plan, working from home) in this process; and also making the assessment of “undue hardship” with concrete criteria such as cost, nature of the work and size of the business can be recommended.
(C) In family law, it may be useful to develop a transparent coefficient system that will contribute to alimony calculations sensitive to the course of the disease; to define review mechanisms at regular intervals and to integrate care or rehabilitation supports into alimony decisions.
(D) In criminal law, practices that take into account the vulnerability of the victim (providing support persons, accessible environments, communication support, testifying according to the fatigue or distraction of the person, or adjusting the hearing time appropriately) can reduce secondary victimizations; for the accused, making neurocognitive assessments with common criteria and basing reports on performance indicators ensures clarity in criminal responsibility assessments and It is thought that it can increase predictability.

### Emerging neurotechnologies in criminal proceedings

Although our dataset predates routine use of advanced neurotechnologies in Turkish criminal practice, the international debate increasingly addresses how emerging neuro-tools (including AI-supported “truth-detection” and neurocognitive assessment technologies) may enter criminal proceedings. Comparative doctrinal analyses under European Court of Human Rights jurisprudence and United States standards highlight that such evidence can raise admissibility challenges and directly engage the privilege against self-incrimination, thereby requiring clear validation thresholds, proportionality, and procedural safeguards when used in capacity-related evaluations [31].

This study has several limitations. First, the analysis is based solely on publicly available and anonymized Supreme Court decision texts; therefore, first-instance judgments and full case files (including the complete content of expert reports) were not accessible. Second, because decision texts often omit detailed sociodemographic and clinical variables (e.g., education, income, disease duration), the observed patterns cannot be interpreted as causal relationships. Third, the absence of explicit references to standardized neurocognitive tests in decision texts does not necessarily mean that such tests were never administered; some evaluations may not have been reflected in the published reasoning. Fourth, differences in publication and redaction practices across chambers and time periods may introduce selection and reporting bias. Fifth, as an appellate-level dataset, the material may not be fully representative of first-instance practice or of disputes resolved without reaching cassation, and MS may be underreported or inconsistently documented in written reasons. Future research could triangulate these findings using first-instance decisions, access to expert-report archives where feasible, interviews with judges or forensic experts, and comparative analyses across neurological conditions. Accordingly, the findings should be interpreted cautiously.

## Conclusion

This study shows that individualized neurological and cognitive assessment in judicial decision processes often lags behind institutional reports and that this is not unique to Turkey. Since similar examples are observed in different countries, it seems to be an important necessity to strengthen judicial standards in an individual-centered manner. Making independent neurocognitive assessment a basic practice, especially in disability cases; defining transparent and measurable “reasonable accommodation” criteria in labor disputes; In criminal law, the clear inclusion of neurocognitive status and victim vulnerability in the reasons for decisions can make national practices more compatible with CRPD principles. The decisions examined generally point to a common picture in which institutional reports are decisive, reasonable regulatory practices are limited, family law shows low sensitivity to long-term economic vulnerability, and neurocognitive assessments are infrequently represented in the criminal field. Incremental and feasible improvements—explicitly requesting independent neurological expertise and functional testing in disability files; making the assessment of “undue hardship” in labor disputes with evidence-based parameters; development of disease-sensitive calculation methods in alimony decisions; and in criminal cases—a reasoned assessment of the victim’s vulnerability and the defendant’s neurocognitive state—can strengthen access to justice and contribute to the establishment of the inclusive approach envisaged by the CRPD.

These proposals should be considered as a roadmap that aims to move step by step within the existing legal and institutional structure, rather than definitive solutions.

## Data Availability

All decision texts analyzed in this study were obtained from the publicly accessible decision search system of the Supreme Court of Turkey (Court of Cassation) (https://karararama.yargitay.gov.tr/).To ensure transparency and reproducibility, an anonymized extraction matrix derived from the reviewed decisions—containing cham-ber/assembly, year, and case type—together with the full codebook and structured search strategy, has been deposited in a public repository (Zenodo): 10.5281/zenodo.18708940. Because the primary sour-ces consist of publicly available judicial texts, no additional raw data are subject to access restrictions. Supple-mentary analytic materials and documentation can be provided by the corresponding author upon reasonable request.
